# Biometric Characterization of the Portuguese Autochthonous Hens Breeds

**DOI:** 10.3390/ani11020498

**Published:** 2021-02-14

**Authors:** Nuno V. Brito, Júlio C. Lopes, Virgínia Ribeiro, Rui Dantas, José V. Leite

**Affiliations:** 1 Center for Research and Development in Agrifood Systems and Sustainability—Polytechnic Institute of Viana do Castelo, 4990-706 Refoios do Lima, Portugal; juliocesar@esa.ipvc.pt; 2 AMIBA—Associação de Criadores de Raça Bovina Barrosã, 4730-260 Vila Verde, Portugal; virginia.ribeiro@amiba.pt (V.R.); rui.dantas@amiba.pt (R.D.); jose.leite@amiba.pt (J.V.L.)

**Keywords:** hens, native breeds, biodiversity, body dimensions, biometric characteristics

## Abstract

**Simple Summary:**

Autochthonous poultry breeds have been forsaken, forgotten even, since they have always been of less importance in the rural socio-economic context, associated with the domestic economy and, above all, regards from a perspective of self-consumption. The study, protection, improvement and dissemination of breeds, has had an almost inexplicable absence of works on the subject with the first reference to Portuguese poultry breeds in the 30’s of the last century. The biometric study of the breeds is fundamental for the knowledge of the morphological characteristics and the productive potentialities. The aim of this study is to characterize the Portuguese hens breeds (“Pedrês Portuguesa”, “Preta Lusitânica”, “Amarela” and “Branca”), using different biometric measures and live weight and to evaluate, under production conditions specific to the artisanal system, the effect of several factors in each of the studied breeds. The results revealed a high sexual dimorphism and that the “Branca” breed stands out in all the biometric measures. Autochthonous Portuguese hens present morphological traits which would made them more prone to meat production (“Branca”), although the dimensions of certain morphological variables could make them suitable for double-purpose production (“Pedrês Portuguesa” and “Amarela”) and is imperative to consider breeding programs that underline their productive potential.

**Abstract:**

Promotion of the conservation and preservation of local breed’s biodiversity combined with the concept of sustainable agriculture and development of economically marginal areas are important policies to implement in modern society. The biometric characterization, contributing to maintain phenotypic traits, is a significant tool in breeding programs, which revaluate local breeds, allow the preservation of animal biodiversity and support consumer demands. This paper approaches the biometric characterization of the Portuguese poultry breeds through the study of sexual dimorphism and breed differentiation using six zoometric measures as differentiation criteria. A total of 429 fowl (66 males and 426 females) were studied and the parameters recorded were body weight, body length, chest circumference, shank length, shank diameter and wingspan. A highly sexual dimorphism was evident, in all breeds, with the “Branca” breed being the most zoometrically distant. Concerning Principal Component Analysis, the highly correlations observed between body length, wingspan and shank length, determined the generalized animal form and could be used as selection criteria for improving body size. Breeding programs aiming to preserve these local genetic resources should consider the dual purpose of these breeds: sustainability and cultural legacy, and the offer to urban consumers a source of differentiated high-quality products.

## 1. Introduction

Animal biodiversity, the grouping of populations into breeds and domestication have evolved over the centuries, from prehistoric times to the present agricultural sedentary lifestyle, a consequence of both natural selection and human intervention. Likewise, the use of these breeds or populations, namely the poultry, for the survival of the Human species, should not be overlooked.

The characterization of animal genetic resources for food and agriculture (ANGR) comprises several types of information: phenotypic, genetic and historical, in the alignment of the Action for Animal Genetic Resources, recognizing that “A good understanding of breed characteristics is necessary to guide the taking decision-making in livestock development and breeding programs” [1,2].

Among avian species, chickens have by far the greatest number of breeds at risk on a global scale. The proportion of avian breeds of unknown risk status is even greater than for mammalian species and extinct breeds have mainly been reported among chickens and the regions with the greatest proportions of breeds classified as at risk for avian breeds are North America, Europe and the Caucasus (48 and 43 percent, respectively) [3].

It is estimated that about 25% of the 1592 chicken breeds [3] are the subject of conservation programs, 40 breeds of chicken have become extinct [2,3,4,5] and only 15% of countries have poultry conservation programs, covering 63% of local breeds and 11% of national populations of transboundary breeds [6]. It urges to maintain programs that could protect breeds in danger of extinction and help to save and spread their genetic diversity, in particular those local breeds that are characterized by medium or low performance and maintained in small populations [7,8].

The breeds’ standardization, based on the morphological classification of racial importance described in the standard (qualitative classification) and its productive characteristics (quantitative classification), proves to be an important instrument for the evaluation of the animal and/or the flocks that constitute the herd existing. This evaluation should be used to identify positive and negative aspects of each animal, in order to determine and promote measures for the selection of animals or flocks [2,9].

The first reference to the Portuguese poultry breeds is recent, in the 30s of the last century. Manuel Véstia, in 1959 [10], differentiated and classified the populations as “Preta Lusitânica” or “Transmontana”, “Pedrês Portuguesa”, “Amarela” or “Galinha do Minho” and “Branca” or “Raça de Pescoço Pelado”. Recent studies of the Portuguese chicken breeds were mainly related to phenotypic and productive characteristics, defining patterns and productive systems [9,11,12].

Portugal, despite being a country of reduced physical dimension, is very different from the orographic, climatic and edaphic points of view. This diversity explains the variety and complexity of the vegetable landscape and has, in turn, resulted in a multiplicity of traditional farming systems in which a high number of indigenous breeds of domestic animals stands out, four of which are birds (chickens) unit [13,14]. Portugal is the European country with the largest number of autochthonous breeds per area unit [14].

Autochthonous hens are produced in a smallholding context, mainly in Northwest Portugal [11,12,13,15]. This territory presents a worrisomely aging population, which resulted from the great wave of emigration during the 1960’s alongside with the continuous attraction for the coastal regions, which has economic, social and culturally shaping the rural landscape of our times.

It is in this region that autochthonous breeds of chickens have their manor, being reared in productive systems complementary to other agricultural activities, considering the production of meat and eggs as by-products of the farm, primarily for self-consumption. Indirectly, these small family farms played an important role in preventing the complete extinction of these breeds [12,13].

Thanks to their affection for what is genuinely Portuguese, these persistent farmers have defended, preserved and carried until today a unique and endangered genetic heritage, which requires the simultaneous intervention of technicians and breeders in its conservation and improvement.

Today, the only four autochthonous chicken breeds are produced in free-range conditions, with a simple, functional and traditional construction, adapted to the number of animals and type of production (meat or eggs) [13,15,16]. The equipment is rudimentary, without much technical or technological evolution. The same rusticity is present in the nutritional aspect—farm fodder and the use of surplus or by-products of human or animal feeding complement the reduced needs of these animals, as well as the search for animal protein (insects, worms), in the very typical and leisurely act of etching the soil [13,14,16].

The sustainability of production is becoming an increasingly strong consumer argument for the choice of products and producers that are part of their diet [17,18,19]. The breeding system for indigenous species is balanced by the use of natural resources, land and water, making it environmentally “friendlier”, and particularly adjusted in less favored regions where these resources are scarce [16,20,21,22,23,24].

Traditionally, the production of local chicken breeds (breeding, fattening and then slaughtering of males) has been for gastronomic purposes, in a generally slow-growing and late-maturing period, especially when grown in systems with reduced inputs. The husbandry practices, in this artisanal system, are characterized by the use of rustic animals in free-range conditions with a low capital investment, in a very efficient productive management to develop purposes of high-biological-value protein such as meat and eggs [25,26,27]. In fact, recent studies confirmed the high quality of the eggs of Portuguese native breeds, matching or superseding the quality of commercial breeds product, an interesting opportunity for the recent specialized market niches [28].

This incalculable genetic value, comparable to any other patrimony, even monuments, needs to be characterized, preserved, promoted, disseminated and valued so that future generations can study and know it for their advantage [2,4,11,13,14]. After a long period lacking actions related to the conservation of local genetic resources, with the Genealogical Register in an early stage, breed characterization studies began to be carried out, under a genetic conservation program for its morphological measures, egg production, growth and reproductive performance, and genetic makeup [11].

The aim of this study was to perform the zoometric characterization of the Portuguese indigenous breeds, evaluating the large existing phenotypic variability in these populations and the different productive factors that may contribute to this variability. Zoometric traits play an important role in the live weight prediction and subsequently in the performance of animal carcasses [29,30], constituting a high potential economic selection criterion, with a significant impact in the paternal line of autochthonous populations.

The knowledge of the zoometric and productive traits will support the implementation of conservation strategies aimed to ensure the survival of low-efficiency local breeds. In addition to being unprecedented, this data is expected to contribute to the creation of an ICAR working group and, a basis to the implementation and validation of poultry breeding strategies.

## 2. Materials and Methods

The trial was carried out in accordance with EU Directive 2010/63/EU; it complied with the Portuguese legislation on animal care (DL n. 113, 7 August 2013), and adhered to the internal rules of the Polytechnic Institute of Viana do Castelo.

### 2.1. Sample Size and Distribution

The whole sample comprised 492 fowl, 426 hens (86.59%) and 66 roosters (13.41%), distributed by the following autochthonous breeds: “Amarela”, 122 animals [(103 Females (F) and 19 Males (M)]; “Branca”, 120 animals (107 F and 13 M); “Preta Lusitânica”, 127 animals (107 F and 20 M) and “Pedrês Portuguesa”, 123 animals (109 F and 14 M).

All animals, over the age of 6 months, were listed in the Genealogical Register of the respective breed and are originated from 19 explorations in the region considered to be the breeding area (districts of Viana do Castelo, Braga and Porto). These farms are characterized by a small number of animals (less than 50 F) divided into several flocks and usually from different breeds. Each flock has, on average, 1 male for every 10 females. Traditionally, the production of autochthonous chickens has been undertaken for double purposes: egg production (hens), and breeding, fattening and slaughtering (roosters), with the ideal slaughter weight being achieved in about 9 to 12 months.

### 2.2. Zoometric Measures

Biometric variables were measured and following procedure, according to FAO (2012) guidelines for adult animals (older than 6 months) [31], is shown in Table 1. Quantitative data was obtained using a digital scale, a gauge with 0.02 mm accuracy, and a measuring tape. Body weight (BW) was estimated using the multifunction scale—KERN HDB with a maximum weight of 5 kg and an interval of 5 g.

### 2.3. Data Analysis

Descriptive statistics [mean, standard deviation (SD), minimum/maximum values] were generated for all the variables in the dataset. The animals were grouped in 3 productive cycles—between 180–360 days old (group 1), from 361 to 720 days old (group 2) and more than 720 days old (group 3). The two-way ANOVA test was used to determine the effects of sex, breed and age group for distinct data categories and differences between means were determined by Tukey’s test using the general linear model analysis of IBM SPSS Statistics 23.0 [32]. All statements of significance were based on testing at the *p*. 0.05 level. The Pearson phenotypic correlation matrix was estimated for BW and zoometric measures (ZM) and principal components analysis (PCA), a method of transforming the original ZM in a new set of orthogonal variables (uncorrelated), called principal components (PC), a linear combination of the original variables, was carried out [33]. The PCA has been used as a tool in the assessment of the body conformation which can be conducted to understand of the complex growth process in the bodily dimensions of an animal during the growth period. Results of principal component analysis not only impact the management of animals but also help in conservation and selection of multiple traits by breeders [34].

## 3. Results

### 3.1. Sexual Dimorphism and Breed Effect

The morphometric analysis indicated highly significantly (*p* ≤ 0.05) sexual dimorphism, as shown in Table 2, with the superiority of the roosters’ weight and zoometric measures. Concerning breed analysis, as for males, the “Branca” breed roosters were significantly (*p* ≤ 0.05) the heaviest, largest and biggest in shank diameter, comparing to the other breeds and presented, as well, a tendency (*p* > 0.05) to a larger chest circumference, a greater shank length and wingspan. Conversely, the “Preta Lusitânica” roosters breed were the significantly (*p* ≤ 0.05) less robust (lighter, shorter, with a smaller chest circumference) males, and presented (*p* > 0.05) the shorter shank length, the smallest shank diameter and wingspan.

No notorious significant differences were observed between hens’ breeds (Table 2), although the “Branca” showed the significantly (*p* ≤ 0.05) highest diameter and shortest shank. The “Amarela” was the shortest (*p* ≤ 0.05) hen and with smallest shank diameter, the “Preta Lusitânica” revealed the smallest chest circumference and the “Pedrês Portuguesa” the largest wingspan.

### 3.2. Age Group Effect

When considering the age group effect (Table 3) in males, significant differences (*p* ≤ 0.05) were observed in the body weight, between all groups, and in the shank diameter, between the first and third groups. In relation to body weight, these results are particularly due to the “Branca” and “Pedrês Portuguesa” breeds’ effect contribution. The same observation was found in the female body weight (Table 4), significantly different between age groups (*p* ≤ 0.05), in particular between the first and the third, in all breeds. A high variability of the body weight was verified, particularly in “Amarela” and “Branca” roosters and “Branca” hens, that could be explained to the recent breeding programs and different practices of production. Additionally, in hens, zoometric modifications were observed (*p* ≤ 0.05), in the measurements of chest circumference and wingspan, with the enlargement of the chest over age, in the” Branca” and “Pedrês Portuguesa” breeds, and, in relation to wingspan, between the first and third groups of the “Pedrês Portuguesa” hen.

### 3.3. Phenotypic Correlations

The phenotypic correlations between linear body measurements and body weight are given in Table 5, for all birds. All the phenotypic correlations, for all the population. between body weight and the body measurements were positive and highly significant (*p* ≤ 0.01) ranging from 0.549 to 0.687. High significant (*p* ≤ 0.01) positive correlations were recorded, for all the animals, between the body length and wingspan (0.76), body weight and chest circumference (0.69), body length (0.66), shank diameter (0.66) and wingspan (0.63).

Similarly, in males (Table 6), high significant (*p* ≤ 0.01) positive relationships were observed for body weight and shank diameter (0.80), wingspan (0.57), chest circumference (0.56) and body length (0.55), and body length and wingspan (0.58). In females (Table 6), significant (*p* ≤ 0.01) high positive relationships were obtained for body weight and chest circumference (0.64), and body length and wingspan (0.59).

### 3.4. Discriminant Analysis

Table 7 presents the eigenvalues, percentage of the total variance along with the rotated component matrix and communalities of the body measurements. The communalities represent estimates of the variance in each variable accounted for by the components. It ranged 0.588–0.867, 0.234–0.835 and 0.440–0.855 in total, M and F respectively. The eigenvalues showed the amount of variance out of the total variance explained by each of the factors.

**Figure 1:**
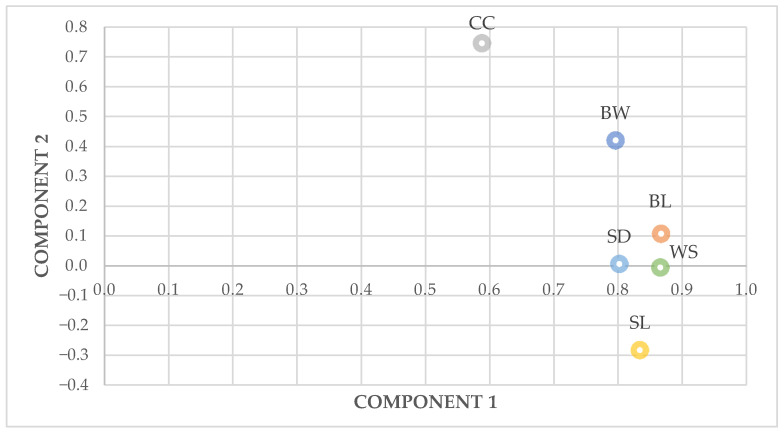
Principal Component Analysis of the zoometric measures in fowls. BW: body weight; BL: body length; CC: chest circumference; SL: shank length; SD: shank diameter; WS: wingspan.

Two principal components were extracted with eigenvalues of 3.949 for the first principal component (PC1) and 0.698 for the second principal component (PC2). The two principal components accounted for 77.4% of the total variance present in the six original variables. PC1 had high loadings on body length (0.867), wingspan (0.866) and shank length (0.834). PC2 was highly correlated with chest circumference (0.747) (Figure 1).

**Figure 2:**
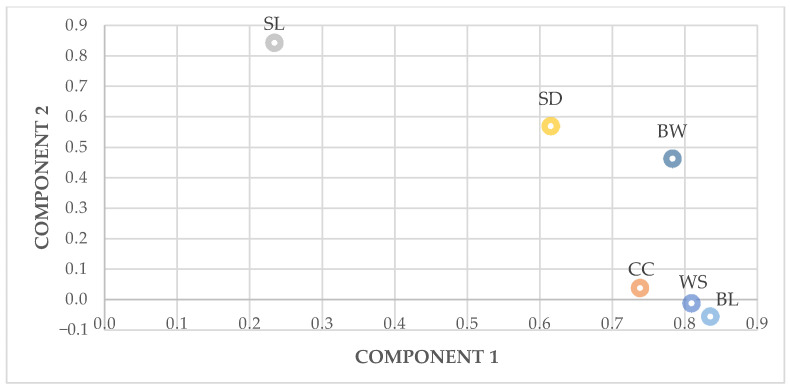
Principal Component Analysis of the zoometric measures in roosters. BW: body weight; BL: body length; CC: chest circumference; SL: shank length; SD: shank diameter; WS: wingspan.

In males, two principal components were extracted with eigenvalues of 3.321 and 0.878 for PC1 and PC2, respectively and accounted for 69.9% of the total variance present in the original variables. PC1 had high positive loadings on body length (0.835), wingspan (0.809) and body weight (0.783). PC2 most highly correlated with shank length (0.844) (Figure 2).

**Figure 3:**
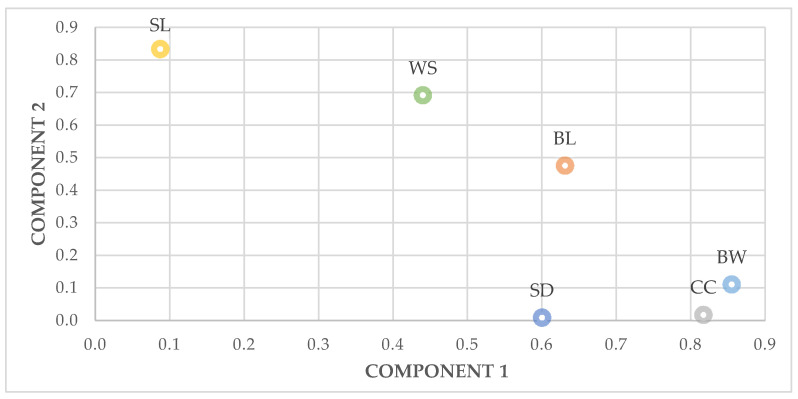
Principal Component Analysis of the zoometric measures in hens. BW: body weight; BL: body length; CC: chest circumference; SL: shank length; SD: shank diameter; WS: wingspan.

In females, the two principal components extracted accounted for 62.8% of the total variance in the original variables with eigen values of 2.809 and 0.809 for PC1 and PC2, respectively. PC1 was most highly correlated with body weight (0.855), chest circumference (0.817) and body length (0.631) and PC2 had high positive loadings on shank length (0.834) and wingspan (0.692) (Figure 3).

## 4. Discussion

Several studies of morphometric index are performed worldwide, the vast majority directly related to the breed characterization and conformation [10,20,24,35,36], but the use of zoometric measures as a strategy to facilitate the implementation of conservation policies aimed to ensure local resources survival, is still beginning in avian populations [37], particularly in Portugal [11,13,38].

The morphometric measurements show highly significant sexual dimorphism, due to hormonal growth effects [39], in accordance with several authors [17,18,19,20,22,24,40,41,42,43,44]. Breed had significant effect (*p* ≤ 0.05) in males, with the superiority of the “Branca” roosters, being the heaviest, largest and with the highest shank diameter and the “Preta Lusitânica” roosters, the lightest, shortest and with the smallest chest circumference.

Concerning the females, no evident differentiations between breeds were observed, although the “Branca” hen presented the largest diameter and shortest shank. The “Amarela” was the shortest hen, indicating the lower potential for egg production, as body and dorsal lengths along with head length are relevant indicators to measure productivity [45,46].

The results observed also reflect the breeds high rusticity and the recent implementation of the selection and breeding programs. Complementary in agricultural production, breed in traditional systems well adapted to the environment, with low nutritional requirements and productivity [20,37,47], these populations, naturally or due to the absence of human intervention, maintained their particular ancestral characteristics [48,49].

The “Branca” revealed to be a strongly built breed, mainly the roosters, and with robust legs. The dimensions of the leg have been related with the type of production, with animals presenting higher dimensions (both in width and length), being more appropriate for meat production and carnic breeds characteristic [44]. On the other hand, “Branca” breeding program began later, only in 2014, and, for decades, its morphological and productive characteristics and color plumage, led to crossbreeding and higher genetic proximity with commercial lines, heavier and with thick hips.

The “Preta Lusitânica” breed was the smallest hen (shorter, lighter, with a smaller chest circumference) hen, reflecting the population with the least productive characteristics, meat and eggs, more used to cultural and religious practices. Without intervention of selection programs and less human action, this breed is genetically closer to the avian populations’ ancestor [12].

Zoometric measures are, generally, stabilized during the first year of age, allowing for the biometric characterization and to contribute to the selection so that breeding programs could be carried out during this period of life. In roosters, the increase of the body weight is accomplished with the strengthening of the shank, and concerning females, beyond the body weight increase, more evident differences were verified in the stronger and robust breeds, “Branca” and “Pedrês Portuguesa”, with chest and wing enlargement. The chest circumference variable proved to be a good indicator of meatiness in most poultry species [45,50].

Morphological traits are essential to implement breeding programs and dimensions of certain morphological variables could make them suitable, from a productive point of view, for meat (breast measurement) or egg productions (body and dorsal lengths, head length) [19,22,24,27,37,51]. Autochthonous Portuguese hens present morphological traits which would made them more prone to meat production (“Branca”), although the dimensions of certain morphological variables could make them suitable for double-purpose production (“Pedrês Portuguesa” and “Amarela”) and is important to consider a breeding programs adjustment to underline their productive potential [36,44,46].

The positive and significant correlations among the body measurements observed in all the groups (total, male and female) indicate high predictability among the variables [34,52,53].

The positive relationship between body weight and the body measurements showed that body weight can be predicted from body measurements in fowls [21,33]. The values of communalities computed for all groups confirm that PCA was appropriate for the data sets and the range of communalities (0.643–0.904; 0.546–0.847; 0.360–0.743) were similar (fowl), slightly inferior (males) or inferior to those reported for body measurements of broilers [39,53,54]. The lower communality observed for shank diameter (0.360) and body length (0.625) in hens interpret the body parameters’ weakness in the body measurements total variation explanation.

PC1 showed that the morphological traits’ variables explain the largest share of total variance, mainly in total and male groups, correlated highly with body length and wingspan, and could be described as “form factor” [39]. In a principal component analysis of body measurements of hens, with PC1 less accounted for the largest variance in the body measurements, high positive loadings on body weight, chest circumference and body length, according to Mendes [54] that reported PC1 high correlation with breast circumference and body weight of Ross 308 broilers. The presence of wide ranges of variation within hens could be explained to the different conditions of human intervention, whether for cultural reasons, in the productive system, or to the agroecological resources adaptation.

## 5. Conclusions

The results revealed the high positive correlations between morphometric measurements and its advantages to define conformation, providing a simple practical methodological framework suited for management, characterization and conservation, to be used in breeding and selection programs.

Portuguese autochthonous poultry breeds are of incalculable ancestral value. These recent selection strategies aim to conciliate the indigenous breeding resistance and adaptation to productive potential. The small size of the population and a strong directional selection may greatly affect the genetic diversity, reinforcing the need for definition and characterization.

Policies that support rural livelihoods, promote local genetic resources and value sustainable products, are a contemporary society requirement. Technical data, such as zoometric measures as a tool in biometric characterization supporting either national or international breeding programs, and scientific strategies is mandatory in the implementation of autochthonous breeds selection programs.

## Figures and Tables

**Table 1:** Biometric variables and measuring procedure to obtain them from the animals.

Variable		How to Measure It
Body Weight (BW)		if spring balance or weigh bridge is available
Body size for adult males and females (to the nearest 0.5 cm)	Body Length (BL)	length between the tip of the rostrum *maxillare* (beak) and that of the cauda (tail, without feathers)
	Chest Circumference (CC)	taken at the tip of the *pectus* (hind breast)
	Shank Length (SL)	length in cm of the shank from the hock joint to the spur of the leg
	Shank Diameter (SD)	length in cm around the shank, near the spur of the leg
	Wingspan (WS)	length in cm between tips of right and left wings after both are stretched out in full

**Table 2:** Effects of sex and sex*breed interaction in the different zoometric measures (cm) and body weight (g) in Portuguese poultry breeds.

		Sex			Breed—Females					Breed—Males				
		F	M	Total	AM	BR	PL	PP	Total	AM	BR	PL	PP	Total
	N	426	66	492	103	107	107	109	426	19	13	20	14	66
BW	Mean	2066 ^a^	2852 ^b^	2172	2050	2116	2065	2033	2066	2911 ^a,b^	3346 ^a^	2547 ^b^	2750 ^b^	2852
	SD	373	578	486	361	413	338	373	373	501	676	468	422	578
	Lower Limit	940	1890	940	940	1070	1290	1220	940	2140	2565	1890	2160	1890
	Upper Limit	3590	5100	5100	2940	3590	2830	3050	3590	3740	5100	3660	3650	5100
BL	Mean	40.4 ^a^	46.1 ^b^	41.2	39.4 ^a^	40.8 ^b^	40.5 ^b^	40.9^b^	40.4	46.2 ^a,b^	47.5 ^a^	45.1 ^b^	46.1 ^a,b^	46.1
	SD	2.2	2.3	3.0	2.5	2.1	2.1	2.0	2.2	2.2	2.8	2.0	1.9	2.3
	Lower Limit	30.1	42.3	30.1	30.1	35.5	35.3	36.3	30.1	42.6	43.6	42.3	43.0	42.3
	Upper Limit	49.3	52.0	52.0	49.3	46.5	46.0	46.0	49.3	51.4	52.0	48.8	48.9	52.0
CC	Mean	33.2 ^a^	36.6 ^b^	33.6	33.0 ^a^	34.0 ^a^	32.2 ^b^	33.1 ^a^	33.2	37.2 ^a^	38.4 ^a^	34.3 ^b^	37.3 ^a^	36.6
	SD	2.7	3.1	3.0	2.9	2.7	2.5	2.6	2.7	3.5	2.2	2.9	1.5	3.1
	Lower Limit	25.3	29.0	25.3	25.3	27.5	26.6	25.8	25.3	30.8	35.0	29.0	35.4	29.0
	Upper Limit	43.5	45.8	45.8	43.5	41.0	38.5	43.4	43.5	45.8	43.0	38.1	40.3	45.8
SL	Mean	5.9 ^a^	7.2 ^b^	6.1	5.9 ^a,b^	5.7 ^b^	6.0 ^a^	6.0 ^a^	5.9	7.2	7.4	7.1	7.3	7.2
	SD	0.5	0.6	0.7	0.4	0.7	0.5	0.4	0.5	0.7	0.5	0.5	0.5	0.6
	Lower Limit	4.0	5.8	4.0	4.8	4.0	5.0	4.8	4.0	5.8	6.4	6.4	5.9	5.8
	Upper Limit	7.6	8.4	8.4	6.8	7.3	7.6	7.0	7.6	8.4	8.3	8.4	8.0	8.4
SD	Mean	13.6 ^a^	16.8 ^b^	14.0	12.8 ^a^	14.3 ^b^	13.9 ^c^	13.3 ^d^	13.6	16.8 ^a^	18.3 ^b^	16.3 ^a^	16.3 ^a^	16.8
	SD	1.3	1.5	1.7	0.9	1.3	1.2	1.1	1.3	1.4	1.4	1.2	1.3	1.5
	Lower Limit	11.2	14.3	11.2	11.2	11.2	11.8	11.2	11.2	14.7	16.2	14.3	15.0	14.3
	Upper Limit	18.5	20.7	20.7	15.3	18.5	17.8	17.8	18.5	19.8	20.7	19.1	19.4	20.7
WS	Mean	48.4 ^a^	56.6 ^b^	49.4	47.8 ^a,b^	47.3 ^a^	48.5 ^b^	49.9 ^c^	48.4	55.5	57.2	55.0	57.3	56.6
	SD	3.0	2.9	4.0	2.7	3.3	5.0	2.7	3.0	2.2	3.7	2.8	2.1	2.9
	Lower Limit	39.8	50.0	39.8	39.8	39.8	41.0	42.5	39.8	52.5	52.2	50.0	53.8	50.0
	Upper Limit	55.8	63.3	63.3	55.8	53.7	54.2	54.8	55.8	61.2	63.3	63.3	60.4	63.3

^a,b,c,d^ Different letters in the superindex are indicative of the existence of significant differences among sex or among sex*breed interaction for the zoometric measures and body weight (*p* ≤ 0.05). If the same letter is present in different sex or sex*breed interaction, within each analyzed measure, then, no significant difference is found. F: female; M: male; AM: “Amarela”; BR: “Branca”; PL: “Preta Lusitânica”; PP: “Pedrês Portuguesa”; N: number; SD: standard deviation; BW: body weight; BL: body length; CC: chest circumference; SL: shank length; SD: shank diameter; WS: wingspan.

**Table 3:** Effects of age group and breed in the different zoometric measures (cm) and body weight (g) in roosters.

		Age			AM			BR			PL			PP		
	Group	1	2	3	1	2	3	1	2	3	1	2	3	1	2	3
	N	35	22	9	12	3	4	4	7	2	9	9	2	10	3	1
BW	Mean	2577 ^a^	3017 ^b^	3523 ^c^	2679	3218	3379	2933 ^a^	3265 ^a^	4460 ^b^	2336	2684	2888	2530 ^a^	3235 ^b^	3500
	SD	350	552	696	443	310	349	276	453	905	195	614	209	184	363	-
	Lower Limit	2120	1890	2740	2140	2860	3080	2700	2565	3820	2120	1890	2740	2160	2980	3500
	Upper Limit	3520	4010	5100	3520	3405	3740	3265	4010	5100	2690	3660	3035	2820	3650	3500
BL	Mean	45.6	46.6	47.1	45.8	46.6	47.4	46.8	47.8	47.9	44.3	45.7	46.3	46.0	46.5	46.2
	SD	2.1	2.1	3.2	1.8	1.3	3.5	2.5	2.4	5.8	1.8	2.0	2.6	2.2	1.5	-
	Lower Limit	42.3	42.5	43.8	42.6	45.7	44.2	44.6	43.6	43.8	42.3	42.5	44.4	43.0	45.5	46.2
	Upper Limit	50.3	51.0	52.0	50.0	48.0	51.4	50.3	51.0	52.0	48.8	48.8	48.1	48.9	48.3	46.2
CC	Mean	36.0	36.8	38.4	36.4	39.6	37.8	37.6	38.2	40.8	33.4	34.8	36.4	37.2	36.9	40.3
	SD	3.3	2.9	2.2	4.1	2.1	0.8	2.1	1.8	3.1	2.9	3.0	1.4	1.2	1.5	-
	Lower Limit	29.2	29.0	35.4	30.8	37.3	36.8	35.0	35.4	38.6	29.2	29.0	35.4	35.4	35.8	40.3
	Upper Limit	45.8	41.5	43.0	45.8	41.5	38.5	39.8	40.0	43.0	37.0	38.1	37.4	39.5	38.6	40.3
SL	Mean	7.2	7.3	7.2	7.1	7.6	7.1	7.5	7.4	7.4	7.0	7.2	7.6	7.2	7.5	7.1
	SD	0.6	0.5	0.6	0.9	0.1	0.3	0.2	0.7	0.9	0.3	0.4	1.2	0.6	0.4	-
	Lower Limit	5.8	6.4	6.7	5.8	7.5	6.7	7.3	6.4	6.8	6.4	6.4	6.7	5.9	7.1	7.1
	Upper Limit	8.4	8.3	8.4	8.4	7.7	7.4	7.7	8.3	8.0	7.5	7.8	8.4	8.0	7.9	7.1
SD	Mean	16.3 ^a^	17.1 ^a,b^	18.1 ^b^	16.3	17.1	17.	17.5	18.4	19.6	16.4	16.1	16.4	15.9	16.8	19.4
	SD	1.1	1.7	1.6	1.4	0.5	1.3	1.1	1.4	1.6	0.9	1.6	0.7	0.7	1.5	-
	Lower Limit	14.7	14.3	15.9	14.7	16.6	16.9	16.2	16.6	18.5	15.0	14.3	15.9	15.0	15.6	19.4
	Upper Limit	18.8	20.7	20.7	18.8	17.5	19.8	18.8	20.7	20.7	17.5	19.1	16.9	17.2	18.5	19.4
WS	Mean	55.6	56.3	57.3	55.3	56.2	55.8	56.2	56.9	60.5	55.0	54.9	55.8	56.3	59.5	60.1
	SD	2.1	3.5	3.4	2.4	0.3	3.0	1.8	4.3	3.6	2.5	3.4	3.5	1.6	1.4	-
	Lower Limit	52.1	50.0	53.3	52.5	56.0	53.4	54.6	52.2	57.9	52.1	50.0	53.3	53.8	57.9	60.1
	Upper Limit	61.2	63.3	63.0	61.2	56.5	60.1	58.5	63.3	63.0	60.2	59.2	58.2	59.3	60.4	60.1

^a,b,c^ Different letters in the superindex are indicative of the existence of significant differences among age group for zoometric measures and body weight (*p* ≤ 0.05). If the same letter is present in different age groups within each analyzed measure then, no significant difference is found. Age 1: 180–360 days; Age 2: 361–720 days; Age 3: >720 days: BW: body weight; BL: body length; CC: chest circumference; SL: shank length; SD: shank diameter; WS: wingspan; AM: “Amarela”; BR: “Branca”; PL: “Preta Lusitânica”; PP: “Pedrês Portuguesa”; N: number; SD: standard deviation.

**Table 4:** Effects of age group and breed in the different zoometric measures (cm) and body weight (g) in hens.

		Age			AM			BR			PL			PP		
	Group	1	2	3	1	2	3	1	2	3	1	2	3	1	2	3
	N	126	206	94	18	56	29	34	37	36	29	61	17	45	52	12
BW	Mean	1911 ^a^	2085 ^b^	2234 ^c^	1781 ^a^	2078^b^	2166 ^b^	2004 ^a^	2056 ^a^	2284 ^b^	1902 ^a^	2098 ^a,b^	2231 ^b^	1898 ^a^	2100 ^a,b^	2253 ^b^
	SD	306	389	337	283	354	345	373	505	283	261	349	321	272	388	479
	Lower Limit	940	1070	1530	940	1360	1530	1390	1070	1710	1290	1435	1675	1375	1220	1690
	Upper Limit	2960	3590	3050	2210	2940	2910	2960	3590	2870	2650	2795	2830	2395	2880	3050
BL	Mean	40.5	40.4	40.4	39.2	39.4	39.4	41.0	40.5	40.8	40.2	40.7	40.2	40.9	40.9	41.5
	SD	2.4	2.3	2.1	2.4	2.8	1.9	2.4	2.0	1.9	2.0	2.1	2.1	2.4	1.7	1.7
	Lower Limit	33.7	30.1	35.6	33.7	30.1	35.6	36.0	35.5	37.0	35.3	35.2	37.0	36.3	36.9	39.3
	Upper Limit	46.5	49.3	45.0	45.3	49.3	43.7	46.5	44.0	45.0	43.9	46.0	44.4	46.0	44.9	44.2
CC	Mean	32.5 ^a^	33.1 ^a,b^	34.1 ^b^	32.6	33.4	33.8	32.9	33.9	35.0	31.8	32.4	32.2	32.8 ^a^	33.0 ^a,b^	34.9 ^b^
	SD	2.6	2.8	2.5	3.8	2.9	1.9	2.5	3.1	1.9	2.1	2.7	2.6	2.	2.5	3.3
	Lower Limit	25.3	26.6	29.2	25.3	27.4	30.2	27.5	28.0	31.4	27.7	26.6	29.2	25.8	27.8	30.2
	Upper Limit	42.9	43.5	43.4	42.9	43.5	36.9	39.3	41.0	39.0	35.5	37.2	38.5	36.2	39.7	43.4
SL	Mean	5.9	5.9	5.8	5.9	5.9	5.8	5.7	5.8	5.7	6.0	6.0	5.8	5.9	6.0	6.0
	SD	0.5	0.5	0.5	0.4	0.4	0.4	0.6	0.7	0.6	0.4	0.5	0.5	0.5	0.4	0.3
	Lower Limit	4.0	4.3	4.3	5.4	5.2	4.8	40	4.3	4.3	5.3	5.2	5.0	4.8	5.3	5.4
	Upper Limit	7.0	7.6	7.2	6.7	6.8	6.7	6.9	7.3	7.2	6.9	7.6	6.7	7.0	6.8	6.3
SD	Mean	13.6	13.5	13.8	12.7	12.8	12.9	14.1	14.2	14.6	14.0	13.8	13.8	13.3	13.2	13.2
	SD	1.2	1.2	1.3	1.0	0.9	0.9	1.2	1.4	1.4	1.2	1.1	1.2	1.1	1.2	0.6
	Lower Limit	11.2	11.2	11.2	11.2	11.2	11.2	11.8	11.1	12.4	12.1	12.1	11.8	11.2	11.2	12.1
	Upper Limit	17.5	18.2	18.5	14.7	15.3	15.3	17.5	18.2	18.5	17.2	17.8	16.2	15.6	17.8	14.0
WS	Mean	48.4 ^a^	48.8 ^a,b^	47.6 ^b^	48.0	48.1	47.4	47.4	47.9	46.6	48.0	49.0	47.5	49.5 ^a^	50.0 ^a,b^	51.4 ^b^
	SD	3.0	2.8	3.4	3.4	2.5	2.6	2.8	3.4	3.5	2.7	2.6	3.5	2.8	2.6	1.7
	Lower Limit	40.5	40.7	39.8	43.2	40.7	39.8	40.5	42.0	39.8	42.3	44.0	41.0	42.5	43.0	48.4
	Upper Limit	55.8	54.8	53.9	55.8	53.1	52.8	52.0	53.7	52.0	53.5	54.2	52.2	54.6	54.8	53.9

^a,b,c^ Different letters in the superindex are indicative of the existence of significant differences among age group for zoometric measures or body weight (*p* ≤ 0.05). If the same letter is present in different age groups within each analyzed measure then, no significant difference is found. Age 1: 180–360 days; Age 2: 361–720 days; Age 3: >720 days; BW: body weight; BL: body length; CC: chest circumference; SL: shank length; SD: shank diameter; WS: wingspan; AM: “Amarela”; BR: “Branca”; PL: “Preta Lusitânica”; PP: “Pedrês Portuguesa”; N: number; SD: standard deviation.

**Table 5:** Pearson’s Correlations between the zoometric measures and body weight for all birds.

	BW	BL	CC	SL	SD
BW					
BL	0.666 **				
CC	0.687 **	0.557 **			
SL	0.549 **	0.590 **	0.377 **		
SD	0.661 **	0.622 **	0.432 **	0.558 **	
WS	0.626 **	0.765 **	0.510 **	0.639 **	0.556 **

BW: body weight; BL: body length; CC: chest circumference; SL: shank length; SD: shank diameter; WS: wingspan. ** Correlation is significant at the 0.001 level (2-tailed). In bold, if high correlated > 0.5.

**Table 6:** Pearson’s Correlations between the zoometric measures and body weight for males (66 M: below diagonal) and females (426 F: above diagonal).

F/M	BW	BL	CC	SL	SD	WS
BW		0.476 **	**0.636** **	0.252 **	0.390 **	0.393 **
BL	0.549 **		0.432 **	0.272 **	0.320 **	0.587 **
CC	0.561 **	0.460 **		0.156 **	0.228 **	0.358 **
SL	0.439 **	0.241	0.249 *		0.175 **	0.364 **
SD	0.797 **	0.449 **	0.390 **	0.396 **		0.193 **
WS	0.573 **	**0.584** **	0.429 **	0.281 *	0.395 **	

BW: body weight; BL: body length; CC: chest circumference; SL: shank length; SD: shank diameter; WS: wingspan. * Correlation is significant at the 0.05 level (2-tailed); ** Correlation is significant at the 0.001 level (2-tailed). In bold, if high correlated > 0.5.

**Table 7:** Eigenvalues and percentage of Total Variance along with the rotated Component Matrix and Communalities of the zoometric measures and body weight of males and females.

		BW	BL	CC	SL	SD	WS	Eigenvalues	% Variance
All	PC1	0.796	0.867	0.588	0.834	0.802	0.866	3.949	65.809
	PC2	0.421	0.108	0.747	−0.282	0.007	−0.005	0.698	11.625
	Communalities	0.811	0.763	0.904	0.775	0.643	0.750		
Male	PC1	0.783	0.835	0.738	0.234	0.615	0.809	3.321	55.346
	PC2	0.463	−0.055	0.038	0.844	0.570	−0.012	0.878	14.625
	Communalities	0.827	0.700	0.546	0.766	0.703	0.655		
Female	PC1	0.855	0.631	0.817	0.087	0.600	0.440	2.809	46.809
	PC2	0.111	0.476	0.018	0.834	0.009	0.692	0.963	16.049
	Communalities	0.743	0.625	0.668	0.703	0.360	0.672		

BW: body weight; BL: body length; CC: chest circumference; SL: shank length; SD: shank diameter; WS: wingspan; PC: principal component.

## Data Availability

The raw data have been submitted to AMIBA (Portuguese Hens Breeding Plan) and are available on request.
